# Inhibition of SIRT2 in merlin/NF2-mutant Schwann cells triggers necrosis

**DOI:** 10.18632/oncotarget.1422

**Published:** 2013-11-15

**Authors:** Alejandra M. Petrilli, Marga Bott, Cristina Fernández-Valle

**Affiliations:** ^1^ Department of Biomedical Science, College of Medicine, University of Central Florida, Lake Nona-Orlando, Florida, USA

**Keywords:** Neurofibromatosis Type2, high-throughput screen, SIRT2, acetylation, merlin, tumor suppressor, AGK2

## Abstract

Mutations in the NF2 gene cause Neurofibromatosis Type 2 (NF2), a disorder characterized by the development of schwannomas, meningiomas and ependymomas in the nervous system. Merlin, a tumor suppressor encoded by the NF2 gene, modulates activity of many essential signaling pathways. Yet despite increasing knowledge of merlin function, there are no NF2 drug therapies. In a pilot high-throughput screen of the Library of Pharmacologically Active Compounds, we assayed for compounds capable of reducing viability of mouse Schwann cells (MSC) with Nf2 inactivation as a cellular model for human NF2 schwannomas. AGK2, a SIRT2 (sirtuin 2) inhibitor, was identified as a candidate compound. SIRT2 is one of seven mammalian sirtuins that are NAD^+^ -dependent protein deacetylases. We show that merlin-mutant MSC have higher expression levels of SIRT2 and lower levels of overall lysine acetylation than wild-type control MSC. Pharmacological inhibition of SIRT2 decreases merlin-mutant MSC viability in a dose dependent manner without substantially reducing wild-type MSC viability. Inhibition of SIRT2 activity in merlin-mutant MSC is accompanied by release of lactate dehydrogenase and high mobility group box 1 protein into the medium in the absence of significant apoptosis, autophagy, or cell cycle arrest. These findings suggest that SIRT2 inhibition triggers necrosis of merlin-mutant MSCs and that SIRT2 is a potential NF2 drug target.

## INTRODUCTION

Neurofibromatosis type 2 (NF2) is a benign tumor disorder of the nervous system caused by mutations in the *NF2* gene that encodes a tumor suppressor called schwannomin or merlin. The hallmark of NF2 is the formation of bilateral schwannomas in the vestibular branch of the auditory nerve. Patients frequently develop additional schwannomas in other cranial, spinal and peripheral nerves as well as meningiomas and ependymomas. Common initial symptoms include hearing loss, dizziness or imbalance; however life-threatening compression of the brainstem also occurs [1]. The choices for NF2 schwannoma treatments are surgical resection or stereotactic radiosurgery. Many schwannomas however are inoperable and surgery often causes complete loss of nerve function, while radiosurgery carries an increased risk of a future secondary malignancy [2]. Currently, a few clinical trials of anti-cancer drugs are underway for NF2 [3, 4]. Molecular studies of merlin's mechanism of action have revealed that merlin regulates signaling from mitogenic, adhesion and extracellular matrix receptors through many essential signaling pathways [5, 6]. However, the pleotropic effect of merlin has made it difficult to identify the most relevant drug targets.

As an alternative approach to drug discovery, we conducted an unbiased high-throughput screen of the library of Pharmacologically Active Compounds (LOPAC) using viability of merlin-mutant mouse Schwann cells (MSC) as a phenotypic assay to identify potential compounds and pathways relevant to NF2 schwannoma treatment. One compound identified in the screen was AGK2, a SIRT2 inhibitor. SIRT2 is one of seven mammalian sirtuins, also known as class III HDACs (histone deacetylases). Sirtuins are NAD(+) dependent deacetylases, that remove the acetyl group from the lysine's epsilon-amine in a multi-step reaction[7, 8]. SIRT2 is mainly cytoplasmic and its known substrates include: α-tubulin, partitioning defective 3 homolog (PAR3), p53, K-RAS, histone H4K16, forkhead Box O1 and 3a (FOXO1 and 3a) and RIP1 [9-14]. While beneficial effects of SIRT2 inhibition was shown in neurodegenerative diseases such as Parkinson's and Huntington's disease, the role of SIRT2 in cancer remains controversial [15, 16]. SIRT2 has been reported to function as a tumor suppressor that is down-regulated in some human gliomas; however, its function has also been reported as essential for survival of C6 glioma cells. Small molecule SIRT2 inhibitors have in some cases selectively induced tumor cell death [17-21].

Here we validate AGK2 as a compound that selectively reduces viability of merlin-mutant MSC compared to normal MSCs. Moreover we demonstrate increased expression levels of SIRT2 in merlin-mutant versus normal MSCs that are associated with a general reduction in lysine acetylation. Phenotypic mechanism of action studies suggests that inhibition of SIRT2 in merlin-mutant SCs triggers a necrotic pathway

## RESULTS

### Merlin-Mutant MSC Have Higher SIRT2 Levels and Lower Lysine Acetylation Levels Than Control MSC

Merlin-mutant mouse Schwann cells (MSC) contain a deletion of exon 2 of the *Nf2* gene that replicates a documented patient mutation. Merlin-mutant MSC were created by *in vitro* adeno-Cre transduction of mouse Schwann cells isolated from sciatic nerves of homozygous *Nf2^flox2/flox2^* mice as previously described [22-24]. Using this NF2 cell model, we screened the Library of Pharmacologically Active Compounds (LOPAC, Sigma-Aldrich) searching for compounds capable of reducing their viability. One of the initial hits we chose to investigate was AGK2, a small molecule SIRT2 inhibitor. The sirtuin family of deacetylases has not been examined heretofore in NF2.

**Figure 1:**
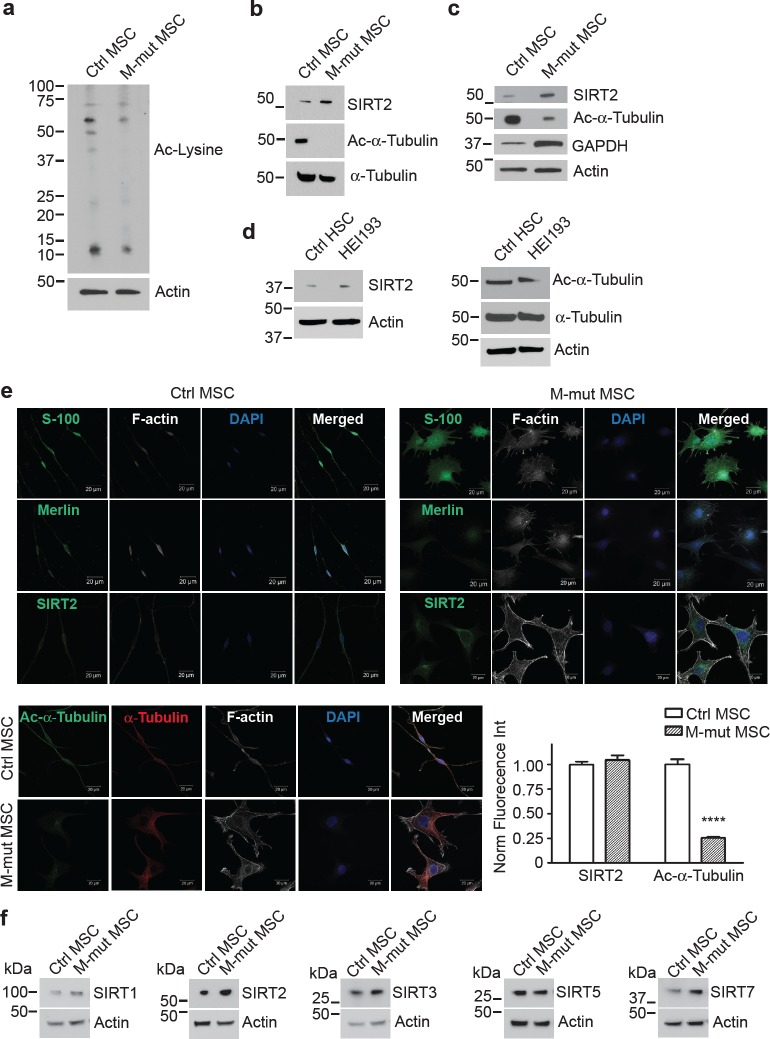
Merlin-Mutant MSC (M-mut MSC) Have Lower Levels of Lysine Acetylation and Higher Levels of SIRT2 Compared to Control MSC Control *Nf2^flox2/flox2^* MSC and merlin-mutant MSC were analyzed by western blotting for: a) Acetyl-lysine, b) SIRT2, acetyl-α-tubulin, α-tubulin and c) SIRT2, GAPDH. Anti-β-actin was used as a loading control. d) Cultured control human Schwann cells (HSCs) from normal individuals and HEI193 cells were analyzed by western blotting for SIRT2, acetyl-α-tubulin, α-tubulin. Anti-β-actin was used as a loading control. e) Representative confocal images of control MSC and merlin-mutant MSC grown overnight on glass coverslips and immunostained with the indicated antibodies (green). The nucleus was visualized with DAPI stain (blue) and F-actin with phalloidin-Alexa633 (white). Scale bar: 20 μm. Quantitation of the immunofluorescence from three independent experiments was performed with Volocity software. ****P<0.0001 determined by two-way ANOVA using Bonferroni post-tests. f) Control *Nf2^flox2/flox2^* MSC and merlin-mutant MSC were analyzed by western blotting for SIRT1, SIRT2, SIRT3, SIRT5, SIRT7 and β-actin.

To visualize the lysine acetylation patterns in merlin-mutant MSC and control MSC, we performed a western blot using an ε-acetyl-Lysine antibody. Control MSC had a higher number of and more intensely acetylated bands than merlin-mutant MSC (Fig. 1a and Supplementary Fig. S1). Because the merlin-mutant MSC presented fewer bands, we asked whether SIRT2, the target of AGK2 had altered expression levels. Indeed we found that merlin-mutant MSC expressed SIRT2 at higher levels than control MSC (Fig. 1b). The acetyl-lysine blot differed greatly around the 50 kDa molecular weight marker. Because SIRT2 is a recognized acetyl-α-tubulin deacetylase, we investigated if any of those bands were α-tubulin. Merlin-mutant MSC have highly deacetylated tubulin levels compared to control MSC (Fig. 1b). We confirmed these differences with several independently derived merlin-mutant MSC lines (Fig. 1c). We next immunoblotted for glyceraldehyde-3-phosphate dehydrogenase (GAPDH) because tumor cells have altered metabolism and sirtuins can influence metabolic and energetic regulation. We found higher levels of GAPDH in merlin-mutant MSC compared to MSC consistent with higher glycolytic activity in merlin-mutant MSC than controls (Fig. 1c). In order to validate the results in our cellular model, we measured SIRT2 and acetyl-α-tubulin levels in a human NF2 cell line created by immortalization of schwannoma cells from a NF2 patient with the E6 and E7 genes of the papillomavirus [25]. Similarly, we found higher levels of SIRT2 in HEI-193 cells than in control human Schwann cells which correlated with lower acetylated tubulin levels in HEI-193 compared to control cells (Fig. 1d).

We localized SIRT2 in control and mutant cells using immunofluorescence staining followed by confocal microscopy. Both cells types expressed the Schwann cell marker S100 and control MSC immunoreacted with the N-terminal merlin antibody whereas the merlin-mutant MSC did not. Both cell types expressed SIRT2 in the cytosol and nuclei, however, merlin-mutant MSC in general had higher levels of fluorescence intensity than controls. Control MSC showed significantly higher levels of acetylated tubulin compared to merlin-mutant MSC and in the latter, the weak immunofluorescence signal for acetylated tubulin was mostly perinuclear (Fig. 1e).

Lastly, we examined the expression of other sirtuin family members by western blotting. SIRT5 was expressed in control and mutant MSC at similar levels, whereas SIRT1, SIRT3 and SIRT7 were expressed at higher levels in merlin-mutant MSC than MSC (Fig. 1f).

### SIRT2 Inhibition Selectively Reduces Merlin-Mutant MSC Viability in a Dose-Dependent Manner

**Figure 2:**
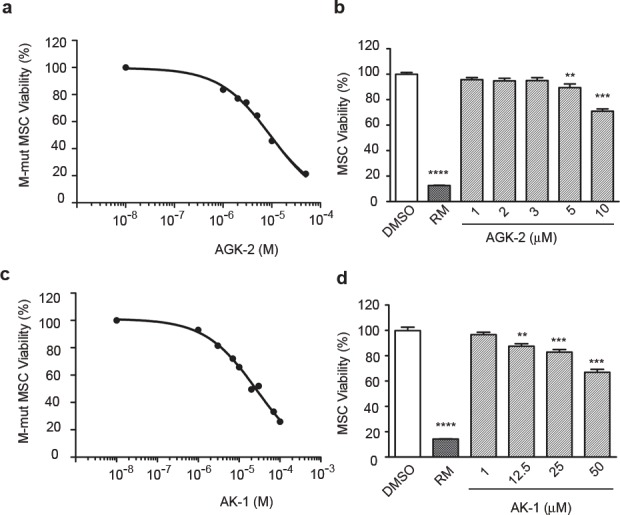
SIRT2 Inhibition With AGK2 and AK1 Selectively Decreases Merlin-Mutant MSC Viability a) AGK2 dose response curve for cell viability. Merlin-mutant MSC were seeded at 5,000 cells/well in 384-well dishes and were incubated with increasing concentrations of AGK2 for 24 hours. Cell viability was assessed with the CellTiter-Fluor assay. DMSO control was considered 100% viability. Rapamycin (RM) (50 μM) was used as positive control for cell death. Graph represents the mean ± SEM of 3 independent experiments analyzed together (n=96), IC_50_=9.01μM, log [inhibitor] vs. response, variable slope (four parameters). b) AGK2 dose response for control MSC viability. Cell viability was measured as in (a). Graph represents the mean ± SEM (n=36). **P<0.01; ***P<0.001;****P<0.0001 determined by one-way ANOVA using Turkey's multiple comparison test. c) AK1 dose response curve for merlin-mutant MSC viability. Cell viability assessed as in (a). Graph represents the mean ± SEM of 3 independent experiments analyzed together (n=96), IC_50_=26.1μM, log [inhibitor] vs. response, variable slope (four parameters). d) AK1 dose viability response for control MSC. Cell viability was measured as in (a). Graph represents the mean ± SEM (n=20). ***P<0.001; ****P<0.0001 determined by one-way ANOVA using Turkey's multiple comparison test.

To evaluate selectivity of SIRT2 inhibition for merlin-mutant MSC viability, we conducted a dose response study of AGK2 using CellTiter-Fluor assay. We found that a 24 hour exposure to AGK2 decreased merlin-mutant MSC viability in a dose-dependent manner with an IC_50_= 9.0 μM (Fig. 2a). In contrast, AGK2 did not decrease control MSC viability as effectively as for merlin-mutant cells (Fig. 2b). At 10 μM AGK2, merlin-mutant cells retained 45.8 ± 0.7 % viability compared to control MSC that retained 70.9 ± 1.8 % viability.

Although AGK2 is a highly selective SIRT2 inhibitor, it does minimally inhibit SIRT1 and SIRT3 at ten times the SIRT2 IC_50_ level when tested *in vitro* [15]. To corroborate that SIRT2 was specifically inhibited by AGK2 and associated with loss of merlin-mutant MSC viability, we tested the effect of an alternative SIRT2 inhibitor, AK1. This benzylsulfonamide also inhibits SIRT2 enzymatic activity by targeting the nicotinamide binding site but with less potency for SIRT1 and SIRT3 than AGK2. We found that AK1 decreased merlin-mutant MSC viability in a dose-dependent manner with an IC_50_= 26.1 μM (Fig. 2c). AK1 minimally reduced control MSC viability which retained 82.8 ± 2.1% viability at 25 μM (Fig. 2d). These results suggest that pharmacological inhibition of SIRT2 selectively reduced merlin-mutant MSC viability.

### SIRT2 Inhibition Reduces Merlin-Mutant MSC Proliferation Without Interfering with Cell Cycle Progression or DNA Synthesis

**Figure 3:**
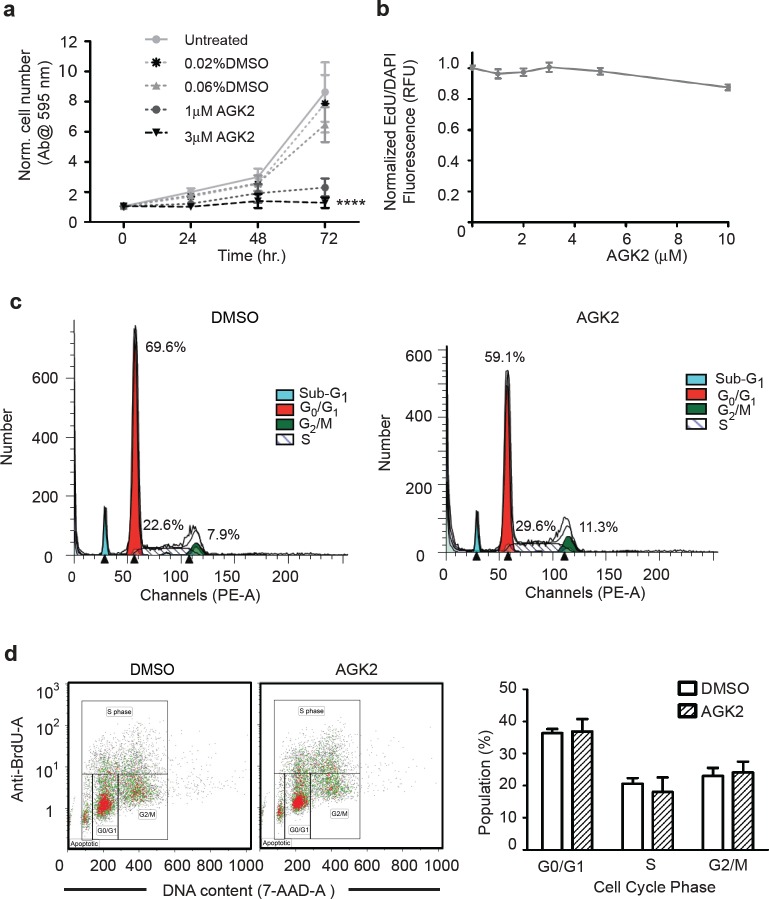
AGK2 Decreases Merlin-Mutant MSC Proliferation Without Interfering With Cell Cycle Progression a) Merlin-mutant MSC were incubated with AGK2 or DMSO for the indicated times and the number of cells was assessed using a crystal violet assay. Graph combines 3 independent experiments in triplicates (mean ± SEM, n=9). b) Merlin-mutant MSC were treated with increasing AGK2 concentrations for 24 hours. For the last 6 hours 10 μM EdU was added to the culture. Incorporation of EdU into S-phase cells was assessed with the EdU Click-It microplate assay. Graph combines 3 independent experiments (mean ± SEM, n=44). c) Merlin-mutant MSC were treated with 5 μM AGK2 for 8 hours prior to propidium iodide labeling. Cells were analyzed by flow cytometry and the diploid cell population was evaluated with the ModFit program. d) Merlin- mutant MSC were treated with AGK2 or DMSO control overnight and with 10 μM BrdU for the last 3 hr of incubation. Cells were analyzed by flow cytometry. Plot of the distribution of BrdU- and 7-AAD-labelled cells analyzed with Kaluza software. These distribution plots are representative of 3 independent experiments (n=3). Bar graph of the distribution of the cell cycle phases of the experiments as mean ± SEM.

We next assessed the ability of AGK2 to decrease cell viability for longer incubation times. We treated merlin-mutant MSC with vehicle or inhibitor and assessed the cell number by crystal violet staining at 24, 48 and 72 hours. We found that at 72 hours AGK2 significantly reduced the number of merlin-mutant MSC compared to vehicle control (Fig. 3a). We also measured EdU incorporation in AGK2-treated merlin-mutant MSC and found that it did not decrease DNA synthesis compared to vehicle treated controls (Fig. 3b).

To determine if the decrease in cell number caused by AGK2 was associated with inhibition of cell cycle progression, we performed flow cytometry analysis of propidium iodine (PI) labeled cells. We found that AGK2 did not significantly alter the distribution of diploid cells in the cell cycle, although there was a tendency to slightly increase the percentage of cells in the G2/M phase (Fig. 3e). To further analyze the effect of AGK2 on cell cycle progression, we performed a BrdU/7-AAD assay. There was no significant change in the distribution of treated and untreated cell populations during the cell cycle after 24 hours (Fig. 3f). These results suggest that SIRT2 inhibition decreased merlin-mutant MSC proliferation by mechanisms independent of interfering with cell cycle progression.

### SIRT2 Inhibition Induces Merlin-Mutant MSC Cell Death

**Figure 4:**
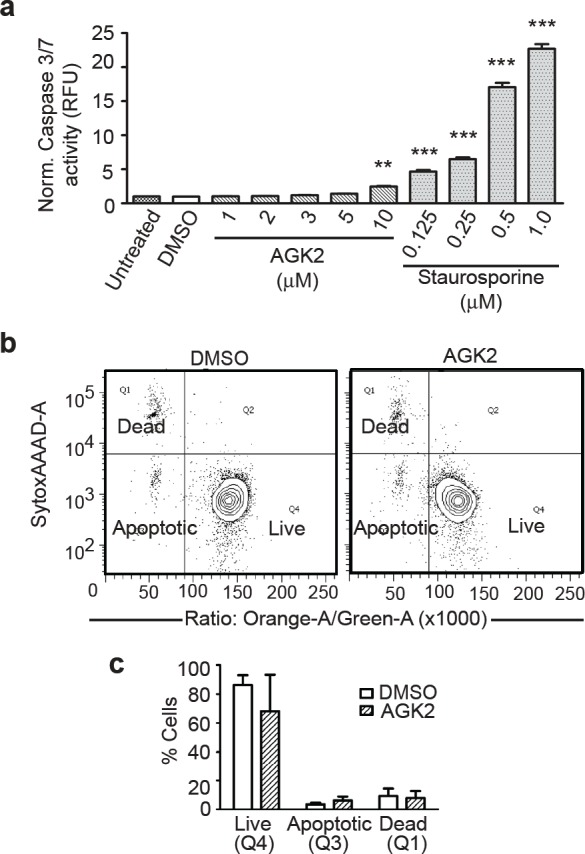
SIRT2 Iinhibition With AGK2 Does Not Induce Significant Apoptosis a) Caspase 3/7 activity assay. Merlin-mutant MSC were seeded in 384 well plates at 5,000 cells/well in 20 μl phenol-red free medium. After 16 hours 5 μl/well of inhibitor/vehicle was added and incubated for an additional 8 hours. Activity of the Caspase 3/7 was measured with the ApoONE Homogeneous assay. Staurosporine curve was used as positive control. Histogram represents 3 independent experiments (n=96) normalized to untreated and analyzed together. **P<0.05; ***P<0.001determined by one-way ANOVA using Dunnett's multiple comparison test to DMSO control. b) Plasma membrane asymmetry was evaluated with the Violet ratiometric assay by flow cytometry. Merlin-mutant MSC were incubated with AGK2 or DMSO vehicle for 24 hours and analyzed by flow cytometry. Densitometry graph: apoptotic (Q3), dead (Q1), live (Q4). Below, bar graph represents mean ± SEM of 3 independent experiments (n=3).

To determine if SIRT2 inhibition reduced merlin-mutant MSC viability by inducing apoptosis, we tested caspase 3/7 activity following AGK2 treatment. We found that AGK2 moderately increased caspase 3/7 activity at 10 μM compared to the positive control, staurosporine, that increased caspase 3 and 7 activity to much higher levels in a dose-dependent manner (Fig. 4a). To further analyze if SIRT2 inhibition induced caspase independent apoptosis, we studied the effect of AGK2 on merlin-mutant MSC membrane asymmetry using the violet ratiometric flow cytometry assay. We found AGK2 only moderately increased apoptosis from 3.5 ± 1.2 % in merlin-mutant MSC treated with DMSO to 6.1 ± 2.8 % when treated with AGK2. This increase however was not statistically significant (Fig. 4b,c). These results suggest that even though AGK2 slightly induced apoptosis it is not the main mechanism responsible for the decreased viability of merlin-mutant MSC.

**Figure 5:**
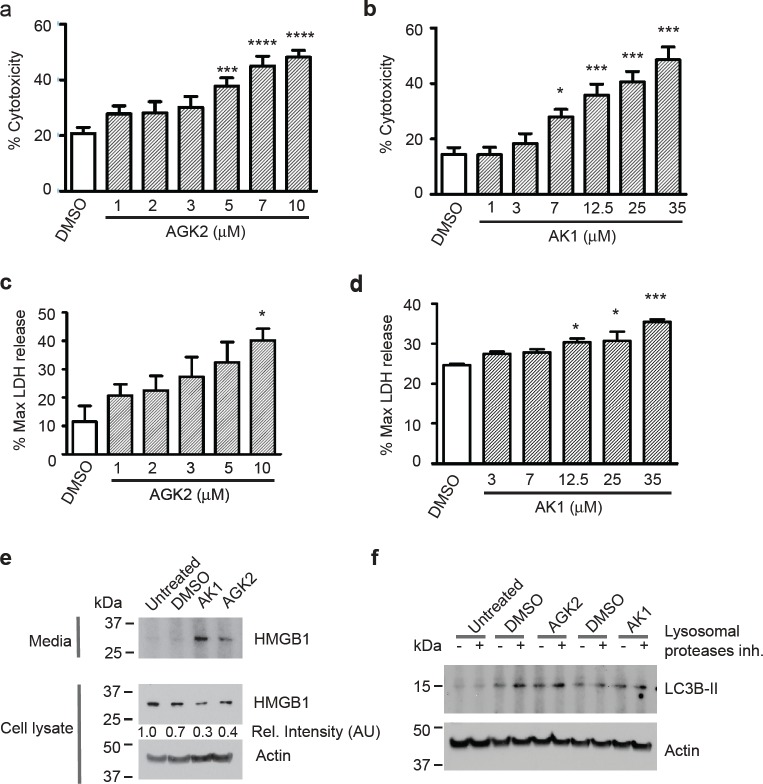
SIRT2 Inhibition Causes Release of LDH and HMGB1 from Merlin-Mutant MSC Merlin-mutant MSC were seeded in 384 well-plates at 5,000 cell/well with cellTox-Green dye in phenol-red free medium. After attachment cells were treated with increasing concentrations of a) AGK2 and b) AK1 and incubated for 24 hours. Cytotoxicity was measure in a plate reader. Digitonin treatment was considered 100% cytotoxicity control. *P<0.05; ***P<0.001; ****P<0.0001determined by one-way ANOVA using Bonferroni's multiple comparison test. c) Merlin-mutant MSC (5,000 cells/well in a 384-well plate) were treated for 4 hours with increasing concentrations of AGK2 and release of LDH into the medium was measured with a fluorogenic assay, the CytoTox-ONE homogenous membrane integrity assay in a plate reader; digitonin was considered as control for maximum LDH release. *P<0.05 determined by one-way ANOVA using Dunnett's multiple comparison test d) Assessment of LDH released to the medium by merlin-mutant MSC treated 4 hours with increasing concentrations of AK1 as in (c), *P<0.05; ***P<0.001 determined by one-way ANOVA using Dunnett's multiple comparison test. e) Merlin-mutant MSC were seeded in 6-well plates and treated for 6 hours with inhibitors/vehicle. HMGB1 release into the medium and cellular content of HMGB1 was assessed by western blot with anti-HMBG1 antibody. Anti-β-actin was used as loading control. f) Induction of autophagy was assessed by LC3B-II immunobloting. Merlin-mutant MSC were treated for 3 hours with AGK2 or AK1 plus and minus 10 μg/ml acidic lysosomal proteases inhibitors (E64-d and pepstatin-A). Cell were lysed in 1% SDS buffer and resolved by western blot for anti-LC3B. Anti-β-actin was used as loading control.

Next, we assessed whether SIRT2 inhibition decreased merlin-mutant MSC viability by inducing cell death through necrosis. We measured cytotoxicity with a fluorescence plate format assay that uses a cyanine dye impermeant to live cells that stains DNA from dead cells and increases its fluorescence. We found that both, AGK2 and AK1, significantly increased the number of dead merlin-null MSC in dose-dependent manners (Fig. 5a,b). To further analyze the mechanism of cell death, we measured the release of lactate dehydrogenase (LDH) from cells with damaged membranes into the medium with the CytoTox-ONE homogeneous membrane integrity assay. We found that both SIRT2 inhibitors, AGK2 and AK1 increased the levels of LDH released to the medium in a dose dependent manner. This is typically associated with cell necrosis (Fig. 5c,d). We also measured release from cells of the necrosis marker high mobility group box 1 protein (HMGB1) by western blot [26]. We found that AGK2 and AK1 induced release of significant amounts of HMGB1 into the medium that corresponded with a decrease in intracellular HMGB1 levels (Fig. 5e). To further characterize merlin-mutant MSC cell death, we assessed the induction of autophagy by immunoblotting for lipidated microtubule-associated protein 1 Light Chain 3 B (LC3B-II). Cytosolic LC3B-I during autophagy is lipidated by Atg 7 and 3 and is converted in LC3B-II that associates with autophagic vesicles [27]. We found that although DMSO slightly activates autophagy, neither AGK2 nor AK1 induced it (Fig. 5f).

These results suggest that SIRT2 inhibition decreased merlin-null MSC viability by triggering cell death characterized by release of LDH and HMGB1.

## DISCUSSION

### SIRT2 and Schwann Cells

SIRT2 was identified as a potential NF2 drug target from a pilot LOPAC screen. We confirmed that SIRT2 protein levels were higher in cultured merlin-mutant MSC compared to cultured control MSC and that SIRT2 activity was essential for survival of merlin-mutant Schwann cells but not wild type MSC. SIRT2 inhibition by AGK2 and AK1 selectively decreased viability of Schwann cells lacking functional merlin. The AGK2 and AK1 dose-dependent loss of cell viability correlated with a dose-dependent cell death that was further characterized as associated with release of the necrotic markers LDH and HMGB1 without induction of autophagy, cell cycle arrest or significant caspase 3/7 dependent or independent apoptosis.

Although SIRT2 has been considered a potential regulator of cell cycle progression [28-30], we did not find evidence for that mechanism of action in this study. SIRT2 was reported to associate with mitotic structures and to increase in abundance and phosphorylation during mitosis. It has been reported to prevent chromosome condensation and entry into M phase in response to mitotic stress. Additionally, overexpression of catalytically inactive SIRT2 increased the number of multinucleated HeLa cells. However in the absence of stressors, SIRT2 activity was not required for cell cycle progression in HeLa and HEK293 cells [31]. In Schwann cells, selective inactivation of *Sirt2* during early embryogenesis did not reduce the number of Schwann cells produced during the period of rapid cell proliferation that occurs at post-natal days 1-5. This supports the conclusion that cell cycle progression in Schwann cells is independent of SIRT2 activity [9] in agreement with our finding that SIRT2 inhibition did not decrease merlin-mutant MSC viability by arresting cell cycle progression but rather by inducing cell necrosis. There is evidence for a role of SIRT2 in necrosis. Nie et al. reported that AGK2 treatment of PC12 cells decreased intracellular ATP levels and increased necrosis without affecting autophagy [32]. Additionally, tumor necrosis factor alpha (TNF-α) was shown to activate necroptosis via deacetylation of receptor-interacting protein 1 (RIP1) by receptor-interacting protein 3 (RIP3) bound SIRT2 allowing the formation of a stable complex in L929 and Jurkat T cells [14]. Due to the prominent role of cytokines in SC development and repair others have shown that while a low concentration of TNF-α induced SC proliferation, a high concentration of TNF-α induced SC growth arrest and apoptosis [33-35]. We have not studied TNF-α in merlin-mutant Schwann cells and additional studies are needed to identify the pathway by which inhibition of SIRT2 activity leads to cell necrosis.

### SIRT2 in Cancer

Similar to our findings in Schwannoma and control Schwann cells, upregulation of SIRT2 mRNA and protein levels has been reported in some cancer cells such as primary acute myeloid leukemia blasts compared to control hematopoietic progenitor cells from healthy individuals [36]. In glioma cells, however, it has been reported that several human glioma samples had reduced SIRT2 mRNA compared to normal brain tissue and that overexpression of SIRT2 decreased HTB14 glioma cells colony formation [18]. Notably, inhibition of SIRT2 activity decreased C6 glioma cells viability [17]. Additionally, aged mice with inactivation of the *Sirt2* gene by deletion of exons 5-8 developed different types of tumors depending on their gender [19]. Treatment of C6 glioma cells with 10 μM AGK2 for 24 hours caused a 60% loss of viability due to both apoptosis and necrosis [17]. Similarly, another SIRT2 inhibitor, compound AC-93253 showed selectively decreasing viability in several human cancer cell lines over their human primary cells controls [21]. The panel included cell lines from prostate, pancreas, cervical and lung cancer. AC-93253 was also selectively cytotoxic to HeLa cells by inducing apoptosis and necrosis. Discrepancies in the positive and negative associations of SIRT2 with tumor development are likely due to differences in cell type, developmental activation patterns for SIRT2 and substrate preferences [9, 37]. For instance histone H4 is a principal substrate for SIRT2 in various cell types but not in oligodendrocytes [11, 38].

### Inhibition of SIRT2 and other Deacetylases

Although AGK2 and AK1 selectively inhibit SIRT2, at higher concentrations they can also inhibit SIRT1 and SIRT3 *in vitro* [15]. Therefore it is possible that at the IC_50_ concentrations used in this study, the compounds could have also slightly decreased SIRT1 and 3 activity. Interestingly, treatment of BCL6-expressing Burkitt lymphoma cells and Burkitt lymphoma xenograft mice with a dual SIRT1 and SIRT2 inhibitor, cambinol, produced a potent antitumor effect [39]. Hence, there may be value to SIRT2 and SIRT1 combinatorial inhibition in schwannoma treatment. A recent study showed that acetylation of K-RAS at lysine104 attenuates its transforming activity in cancer cells and both SIRT2 and HDAC6 deacetylate K-RAS [12]. Moreover, α-tubulin acetylation and cortactin are regulated by SIRT2 and HDAC6 [10, 40, 41]. Due to the increasing interest of HDAC inhibitors in cancer, a new pan-HDAC inhibitor AR42 was tested on merlin mutant schwannoma and meningioma cells. Both *in vitro* and *in vivo* treatment showed a selective anti-proliferative effect on schwannoma and meningioma cells warranting further clinical evaluation for NF2 related tumors [42-44]. AR42, similar to other HDAC inhibitors also decreased Akt phosphorylation, a pathway upregulated in many cancers and NF2. Chen et al. have shown that AR42 targets HDAC1 and HDAC6 by disrupting their HDAC-protein phosphatase 1 (PP1) complexes, which leads to increased PP1-Akt association, and facilitates PP1-mediated dephosphorylation of Akt [45]. Therefore combinatorial inhibition of SIRT2 and HDAC6 could be evaluated when considering modulation of acetylation signaling for NF2 treatment.

We speculate that SIRT2 pharmacological inhibition may have some therapeutic value for NF2-associated schwannomas by promoting necrosis. However, additional research is needed to understand the relationship between merlin and SIRT2 in normal Schwann cells and the effect of merlin inactivation on protein acetylation and cell survival.

## MATERIALS AND METHODS

### Inhibitors

The SIRT2 inhibitors AGK-2; CAS name 2-Cyano-3-[5-(2,5-dichlorophenyl)-2-furanyl]-N-5-quinolinyl-2-propenamide and AK1; CAS name 3-(azepan-1-ylsulfonyl)-N-(3-nitrophenyl) benzamide were purchased from Sigma-Aldrich and Cayman Chemicals. Rapamycin and Staurosporine were from Santa Cruz Biotechnology (Santa Cruz, CA).

### Antibodies

β-Actin (8H10D10) and α-tubulin (DM1A) mouse mAb, Merlin (D1D8), acetylated-lysine (A-K^2^-100), Acetyl-α-tubulin (Lys40) (D20G3)XP, SIRT1(D1D7), SIRT3(D22A3), SIRT5(D8C3), SIRT7(D3K5A) and LC3B(D11)XP rabbit mAb, HMGB1 rabbit Ab were purchased from Cell Signaling (Danvers, MA). GAPDH mAb was from Millipore. SIRT2 rabbit Ab was from Sigma-Aldrich. S-100 rabbit Ab was purchased from Dako Cytomation (Glostrup, Denmark). Secondary antibodies, peroxidase-conjugated goat anti-mouse IgG and goat anti-rabbit-IgG, were purchased from Pierce, Thermo Fisher (Rockford, IL). Goat anti-rabbit-IgG Alexa Fluor488-and -Fluor546 conjugated antibodies were purchased from Invitrogen (Grand Island, NY).

### Mouse Schwann Cell Cultures

MSC were cultured on 200 μg/ml poly-L-lysine hydrobromide (PLL, Sigma-Aldrich), and 10 μg/ml Laminin (Invitrogen) coated 60 or 100-mm Corning dishes. MSC growth medium: DMEM:F12 1:1 (Gibco) plus 1X-N2 supplement (Gibco), 2μM forskolin, 10 ng/ml neuregulin and 1% Penicillin/Streptomycin (Gibco).

### Merlin-Mutant Mouse Schwann Cell Culture

Merlin-mutant MSC generated in the lab were cultured in CellBIND-Corning 100 mm dishes. Merlin-mutant MSC growth medium was DMEM/F12 1:1 (Gibco); 1X-N2 supplement (Invitrogen) and 1% Penicillin/Streptomycin. All protocols are in accordance with guidelines of and approved by the University of Central Florida (UCF) Institutional Animal Care and Use Committee (IACUC). UCF vivarium is International-certified by the Association for Assessment and Accreditation of Laboratory Animal Care.

### Human Schwann Cell and HEI193 Cell Cultures

Vials of frozen human SCs were thawed and seeded on coated 60 mm Corning plates (200 μg/ml PLL and 50 μg/ml Laminin) containing D10M growth medium: DMEM (Gibco) plus 10% heat inactivated fetal bovine serum (HIFBS, HyClone, Logan, UT), 2 μM Forskolin (Sigma), 0.02 mg/ml Pituitary Extract (Biomedical Tech. Inc) and 1% Penicillin/Streptomycin (Gibco). HEI193 cells were purchased from ATCC (Manassas, VA). HEI193 growth medium: DMEM plus 10% HIFBS, 1% Penicillin/Streptomycin.

### Western Blot Analysis

SCs were lysed in modified RIPA buffer (25 mM Tris-HCl pH 7.6; 150 mM NaCl: 1% Triton X-100, 1% Sodium dodecyl sulfate (SDS) with protease inhibitor cocktail and phosphatase inhibitor cocktails 2 and 3, Sigma-Aldrich). To remove cell debris, lysates were centrifuged at 15,000 rpm for 10 min at 4°C. Protein concentration of the supernatant was determined with the DC Assay (BioRad, Hercules, CA). 10–15 μg of sample protein was resolved in 4–20% polyacrylamide gels (Pierce), transferred to a polyvinylidene fluoride (PVDF) membrane (Immobilon-P, Millipore, Bedford, MA), blocked with 5% BSA and incubated overnight at 4°C with anti- acetylated-lysine (1:500), Acetyl-α-tubulin (Lys40) (1:1,000), α-tubulin (1:1,500), SIRT1(1:500), SIRT2 (1:500), SIRT3(1:500), SIRT5(1:500), SIRT7(1:500), HMGB1 (1:500) for cell lysates and at (1:250) for concentrated medium, GAPDH (1:10,000), LC3B (1:500) and β-Actin (1:15,000) primary antibodies, followed by their corresponding secondary antibodies at 1:20,000. Quantification of western blots was done by densitometry using NIH ImageJ software.

### Immunofluorescence

Control and merlin-mutant MSC were plated on coated German glass coverslips (200 μg/ml PLL and 10 μg/ml Laminin) and immunostained as previously described [24]. Images were acquired using a Zeiss LSM710 Confocal microscope with 3 spectral detection channels, 5 laser lines - 458, 488, 514,543 & 633 nm, FL filter set 49 DAPI, EX G365, FL filter set 43 CY 3, FL filter set 38 Endow GFP, all shift free, EC Plan-Neofluar 40x/1.3 DIC WD=0.21 M27 objective lens, with the ZEN2009 software. Fluorescence signals were acquired on separate channels with identical parameters for each labeled protein from a single plane. Fluorescence intensity of the green channel was done using Volocity software. Pictures were processed with the ZEN2011 software in the same manner.

### Cell Viability Assay

Viability of dose-response assays was assessed with the CellTiter-Fluor cell viability assay (Promega) following manufacturer's specifications. Normal MSC and merlin-mutant MSC were seeded at 5,000 cells/well in 20 μl of phenol-red free growth medium in 384-well plates (black with clear bottom, CellBIND-Corning) and centrifuged 1 min at 500 rpm. Cultures were incubated at 37°C, 7% CO2 until attachment (2.5–3.5 hours), then 5 μl of compound/vehicle solution was added to each well. Plates were quick spun again and returned to the incubator for 24 hours. Fluorescence was read with a Synergy H1 Hybrid plate reader (BioTek, Winooski, VT).

### Proliferation Assays

For the 72 hours cell proliferation study, cell numbers were assessed with the Crystal Violet Assay as previously described [46]. Equal number of merlin-mutant MSC were seeded in 24-well plates and cell number was evaluated at 0; 24; 48 and 72 hour time points. Absorbance at 595 nm was measured with a μQuant plate reader (BioTek).

Rate of DNA synthesis was evaluated at 24 hours with the Click-iT EdU Microplate Assay (Invitrogen/ Life Technologies) as previously described [23]. Oregon green-488 and DAPI fluorescence was measured with a Synergy H1 hybrid plate reader.

### Cell Cycle Analysis

The cell cycle was analyzed by flow cytometry with propidium iodine (PI) staining and BrdU/7AAD. PI staining was performed on ethanol fixed cells using PI/RNase solution from Beckton-Dickinson and studied on a BD Canto-II flow cytometer. Histograms were analyzed with ModFit LT software (Verity Software House, Topsham, ME). For each sample, 10,000 events were collected and the diploid population was distributed across G_0_/G_1_, S and G_2_/M phases.

The BrdU/7AAD assay kit was purchased from Beckton-Dickinson. Cells were seeded in 6-well plates and treated overnight with inhibitor/vehicle. On the next day, 10 μM BrdU was added to the cultures for 3 hours, cells were harvested, fixed and analyzed following manufacturer's instruction. The BD Canto-II flow cytometer with the BD FACSDiva™ 6.1.3 software was use for acquisition and Kaluza 1.2 (Beckman Coulter) software for data analysis.

### Apoptosis Assays

For caspases dependent apoptosis the caspase activity was assessed by the Apo-ONE homogeneous Caspase-3/7 assay (Promega) following manufacturer's specifications. For caspases dependent and/or independent apoptosis the membrane asymmetry was measured with the Violet Ratiometric Membrane Asymmetry Probe/Dead Cell Apoptosis Kit (Invitrogen/Life Technologies). Both assay were previously described [23].

### Cytotoxicity Assays

Cytotoxicity was assayed in a 384 well plate format with 5,000 cells/well after 24 hours incubation with the inhibitors/vehicle with the CellTox Green assay (Promega), following the express, no-step addition at seeding method described by the manufacturer. Cells treated with 27 μg/ml digitonin were considered 100% cytotoxicity control. Fluorescence (Ex= 510nm/Em= 532 nm) was measured on a Synergy H1 Hybrid plate reader.

### LDH Release Assay

LDH released into the culture medium was measured with the CytoTox-ONE assay (Promega). Merllin-null MSC were seeded at 4,000 cell/well in 384 well plates and were treated on the next day with increasing doses of inhibitor/vehicle and incubated for 4 hours. Positive 100% cytotoxicity control was 27 μg/ml digitonin. Plate and assay reagent were equilibrated to room temperature for 25 min and after addition of 25 μl of assay reagent, the plate is incubated at room temperature in the Synergy H1 Hybrid plate reader. No stop solution was used. Fluorescence (Ex=560 nm/Em= 590nm) was measured 5 min after the first column received reagent.

### HMGB1 Release Assessment

Equal number of merlin-null MSC per well in CellBIND 6-well plates were cultured overnight. On the next day the medium was replaced with 2 ml treatment medium containing SIRT2 inhibitors/vehicle in duplicate wells and incubated for 6 hours at 37°C, 7% CO2. Then working on ice, media from duplicates were pooled and transferred to cold 15 ml conical tubes, centrifuged for 5 min at 800g, 4 °C. The supernatant (~4ml) was syringe filtered with 0.45 μm filter (Fisher), transferred to cold 7 ml 9K MWCO Pierce concentrators (Thermo Scientific) and centrifuged for 30 min at 4,000g, 4 °C. From 120 to 150 μl of concentrated proteins were obtained and prepared for SDS-PAGE. When media was removed from cultures, the plates were washed once with ice cold PBS and cell were lysed with the modified RIPA buffer mentioned above; protein concentration was measured with the DC assay (BioRad). Equal volume of concentrated proteins from medium and 10 μg of protein of cell lysates were analyzed by western blotting for HMGB1.

### Statistical Analysis

GraphPad Prism version 5.0 for Windows (GraphPad, La Jolla, CA, USA) was used for statistical analysis and graph generation. AGK-2 and AK1 dose-response experiments were analyzed by non-linear regression (four parameters). Experimental data from three independent experiments were statistically analyzed by one or two-way ANOVA with post-tests, as indicated for each experiment.

## Supplemental Figure


