# Supplementary material for: Inhibition of SIRT2 in merlin/NF2-mutant Schwann cells triggers necrosis
Source: Oncotarget. 2013 Nov 15;4(12):2354–65. doi: 10.18632/oncotarget.1422 (PMC3926832; doi:10.18632/oncotarget.1422)
Supplement: Supplementary file 1 [file oncotarget-04-2354-s001.pdf]

Inhibition of SIRT2 in merlin/NF2-mutant Schwann cells triggers necrosis - Petrilli et al

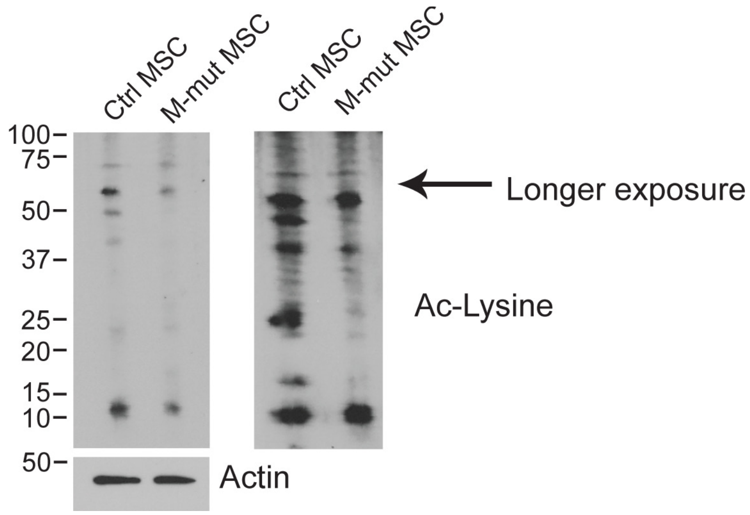

Supp. Fig. 1: Merlin-Mutant MSC Have Lower Levels Of Lysine Acetylation Compared To Control MSC. Cell lysates of control *Nf2*$^{flox2/flox2}$ MSC and merlin-mutant MSC were analyzed by western blotting for acetyl-lysine; β-actin was used as loading control. The right panel shows a longer exposure of the blot and reveals multiple differentially acetylated bands.
